# Characterization of 13 microsatellite loci developed from *Meconopsis horridula*

**DOI:** 10.1590/S1415-47572010005000066

**Published:** 2010-09-01

**Authors:** Yu Zhao, Shi-Bao Zhang, Jing Yang, Lu Zhang

**Affiliations:** 1 College of Landscape and Art, Jiangxi Agricultural University, Nanchang, JiangxiP.R. China; 2 Xishuangbanna Tropical Botanical Garden, Chinese Academy of Sciences, YunnanP.R. China; 3 Germplasm Bank of Wild Species, Kunming Institute of Botany, Chinese Academy of Sciences, Kunming, YunnanP.R. China

**Keywords:** genetic structure, heterozygosity, *Meconopsis horridula*, microsatellite markers, polymorphism

## Abstract

*Meconopsis horridula* is one of the eight most famous flowers in Chinese province of Yunnan. In this study, a modified biotin-streptavidin capture method was used to detect 13 microsatellite markers in the genome of *M. horridula*. The polymorphism of each locus was assessed in 24 samples collected from four populations. The number of alleles per locus ranged from 2 to 7 (mean: 3.2). The observed and expected heterozygosities ranged from 0.0833 to 0.9167 and 0.0816 to 0.8050, respectively. Additionally, nine of the 13 microsatellite markers were successfully amplified in three other congeneric species. These polymorphic SSR markers could be useful for studying the population genetics of *M. horridula* and for assessing genetic variation in this and congenerc species in conservation programs.

*Meconopsis*, an endangered genus of ornamental flowers belonging to the poppy family Papaveraceae, consists of 43 species (Sulaiman and Babu, 1996). *Meconopsis* species have attracted the attention of botanists and horticulturalists because of their beautiful flowers. Some species of *Meconopsis* have also been used as traditional herbal medicine because of their anti-inflammatory and analgesic activities (Samant *et al.*, 2005). *Meconopsis horridula*, a perennial herb with sharp spines on its leaves and stems, is found on grassy or rocky slopes at altitudes of 3000-4900 m in southwestern China. This species is prized as an ornamental and medicinal plant, and has long been used in Chinese traditional herbal medicine because of its anti-inflammatory and analgesic activities (Wang *et al.*, 2003). The low rate of seed germination and seedling recruitment, together with extensive habitat destruction and intensive harvesting, has reduced this species distribution to a narrow range with small populations (Sulaiman and Babu, 1996; Sulaiman and Hasnain, 1996). To date, no nuclear microsatellite primers have been reported for *M. horridula*. In this work, 13 microsatellite markers for this species were developed and characterized. These markers should be useful for studies on the genetic diversity and population structure of *M. horridula*.

A microsatellite-enriched library was prepared using a modified biotin-streptavidin capture method (Chen *et al.*, 2008). Genomic DNA from *M. horridula* samples was extracted from silica-gel-dried leaves using CTAB (Doyle and Doyle, 1987). Total genomic DNA (~ 500 ng) was completely digested with *Mse*I restriction enzyme (New England Biolabs), and then ligated to a specific *Mse*I AFLP adaptor. A digestion-ligation mixture (diluted 1:10) was used for the amplification reaction with the adaptor-specific primer 5'-GATGAGTCCTGAGTAAN-3'. The amplified DNA fragments (200-800 bp) were hybridized to a 5'-biotinylated [(AG)_15_, (AAG)_10_ or (AC)_15_] probe and then selectively separated and captured with streptavidin-coated magnetic beads (Promega) (Zane *et al.*, 2002). The enriched fragments were amplified again with adaptor-specific primers. The polymerase chain reaction (PCR) products were purified with an EZNA gel extraction kit (Omega Bio-Tek) and then ligated into the vector pGEM-T (Promega) before transformation in DH5α cells. Positive clones were picked and tested using the (AAG)_7_/(AC)_10_/(AG)_10_ primers and the vector primer SP_6_/T_7_. Two hundred and fifty-six clones with positive inserts were sequenced with an ABI PRISM 3730XL DNA sequencer. Of these, 140 clones (55%) contained microsatellite sequences, and 70 primer pairs were predicted to be suitable for primer design using the program Primer Premier 5.0 (Clarke and Gorley, 2001).

The presence of polymorphisms in the 70 microsatellite loci was assessed in 24 samples of *M. horridula* collected from four natural populations in Shangri-La of Yunnan Province, their geographic location were 27°51' N 99°43' E, 27°54' N 99°38' E, 27°46' N 99°38' E and 28°08' N 99°54' E, respectively. The microsatellite loci were amplified in a final reaction volume of 20 μL containing 9.5 μL of 2 x Taq PCR MasterMix (Tiangen; 0.1 U Taq polymerase/μL, 0.5 mM dNTP each, 20 mM Tris-HCl, pH 8.3, 100 mM KCl, 3 mM MgCl2), 0.5 μM of each primer and 100-200 ng of genomic DNA. PCR amplifications were done under the following conditions: 97 °C for 3 min followed by 32 cycles at 94 °C for 1 min, at the annealing temperature of each primer (optimized for each locus; Table 1) for 40 s and 72 °C for 40 s, and a final extension at 72 °C for 5 min. The amplified products were then separated on 8% polyacrylamide gels in denaturing conditions and visualized by silver staining. A 20 bp DNA ladder (Fermentas) was used as a standard for molecular size.

The PCR products obtained with the 13 primer pairs revealed polymorphisms among the 24 individuals from the four populations (Figure 1). Standard genetic diversity parameters, departure from Hardy-Weinberg equilibrium and linkage disequilibrium between pairs of loci were estimated with the program GENEPOP v. 4.0 (Raymond and Rousset, 1995). The number of alleles per locus ranged from 2 to 7 (mean: 3.2), and the observed and expected heterozygosities ranged from 0.0833 to 0.9167 (mean: 0.4423) and 0.0816 to 0.8050 (mean: 0.4581), respectively (Table 1). Of the 13 microsatellite loci, MA15, MC44, MC17, MC19 and MC26 deviated significantly from Hardy-Weinberg equilibrium (p < 0.01); there was no significant linkage disequilibrium between locus pairs except for MC44/MC17 and MC17/MA9. The cross-reactivity of the 13 primer pairs was examined in the congeneric species *Meconopsisintegrifolia*, *Meconopsis forrestii,* and *Meconopsis horridula Hook.f.et Thoms.* Amplification products were obtained for nine of the markers (MG36, MC44, MC17, MC19, MG91, MC7, MG56, MG69 and MC90) in all three species.

**Figure 1:**
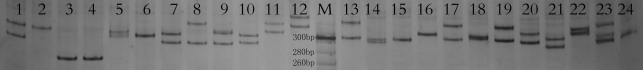
SSR (Simple Sequence Repeats) profile of the MC26 locus in 24 specimens of *M. horridula*. Lane M: 20 bp DNA ladder, Lanes 1-24: samples.

The primers for microsatellite amplification described here may be useful for studying the population genetic structure of *M. horridula* and for assessing genetic variation among populations of *M. horridula* and congeneric species in conservation programs.

## Figures and Tables

**Table 1:** Characteristics of the 13 microsatellite loci developed for *M. horridula.*

Locus	Repeat motif	Primer sequences (5'-3')	Ta (°C)	Allele size (bp)	A	H_O_	H_E_	HWE p value
MA15	(GAA)_12_GTA(GAA)_3_	F 5' GGAAATCAGACATCTCCA 3' R 5' CTTCTCCCGTGTTACTTG 3'	55	170-197	4	0.2500	0.5470	0.0008*
MG36	(CT)_10_	F 5' ATTCAGCAGACAAAGATT 3' R 5' GAACAACCCCATGTAATC 3	55	259-280	4	0.4167	0.6064	0.0257
MC44	(AC)_6_	F 5' CTCACCAATTTGCACTCG 3' R 5' CTTGGAGCCAACTCTTCT 3'	55	185-197	2	0.8333	0.4964	0.0006*
MC25	(AC)_5_	F 5' AGGGCAAGCCAACAGTCA 3' R 5' GGAAGAGGGGCGGTTATA 3'	55	158-165	3	0.7083	0.5709	0.2607
MC17	(GAA)_22_	F 5' CTCAACTTCGTTTCTCGT 3' R 5' CGATGGAAAAGTGGGTAC 3'	55	329-370	4	0.2500	0.5732	0.0015*
MC19	(CT)_2_CC(CT)_7_	F 5' GGGTCGGAACTTGAGGTG 3' R 5' AGAAAGGGAGGGCGAAGG 3'	55	192-200	4	0.9167	0.6055	0.0016*
MC26	(TTC)_4_TA(CTT)_20_	F 5' CAATGAAAAGGAAGAATAAG 3' R 5' AACCAATAGGGCCAGATA 3'	52	280-335	7	0.5417	0.8050	0.0001*
MG91	(AG)_10_	F 5' AGGTTCGGTGAAGTAGCC 3' R 5' TGCCCAGAGCTGAGAAGA 3'	57	200-217	4	0.6667	0.5168	0.4710
MC7	(CT)_4_CG(CT)_5_	F 5' GTTTAGGAGGAACCATTG 3' R 5' TTACTCAGCAAACAGGAA 3'	52	298-310	2	0.1250	0.2528	0.0505
MG56	(CT)_7_	F 5' CTGATTTCTTCCCATTCT 3' R 5' TCTATTTAGTTTCTTGGTG 3'	55	153-170	2	0.5833	0.4889	0.4198
MG69	(AG)_6_	F 5' GTCTTTCTGAATGCTGAG 3' R 5' ATGAGTTTTAGGCCGTTG 3'	55	152-161	2	0.2917	0.2544	1.0000
MA9	(TTC)_6_	F 5' CACCCGCTCATTCTATTC 3' R 5' ACGAACTTCCACTTGCTT 3'	53	195-220	2	0.0833	0.0816	1.0000
MC90	(GA)_10_	F 5' AAGGAGTTCGGATGGATT 3' R 5' CGCCGTTATGTATTAGTT 3'	55	113-120	2	0.0833	0.1560	0.1285

Ta: PCR annealing temperature, A: number of alleles per locus, H_O_: observed heterozygosity, H_E_: expected heterozygosity. *H_O_: significantly different from H_E_ under Hardy-Weinberg equilibrium (HWE) (p < 0.01).
